# Arm lymphedema after vascularized lymph node harvest following Covid-19 vaccination

**DOI:** 10.1080/23320885.2024.2342332

**Published:** 2024-04-17

**Authors:** Tabea Breckwoldt, Pia Niggemann, Lisanne Grünherz, Andrea Weinzierl, Nicole Lindenblatt

**Affiliations:** Department of Plastic Surgery and Hand Surgery, University Hospital Zurich, Zurich, Switzerland

**Keywords:** Vascularized lymph node transfer, Lymphedema, Covid- 19, mRNA vaccination, Lymph node harvest, Donor site lymphedema

## Abstract

There is evidence that COVID-19 vaccines may affect the lymphatic system. We report a case of a 40-year-old female who had undergone lymph node transfer for treating primary lymphedema of the legs. Six months later, the patient developed lymphedema of the right arm closely related to mRNA vaccination against COVID-19.

## Introduction

In response to the Covid-19 pandemic new vaccines such as mRNA vaccines have been applied in many cases following accelerated approval procedures. Long-term effects regarding their interaction with the human organism are to be observed. After widespread use of mRNA vaccines during the pandemic, several side effects related to the lymphatic system have been reported and have sparked further controversy. mRNA application leads to a strong immune reaction in the lymph nodes downstream the injection site, and lymphadenopathy after injection is commonly described as a side effect of the vaccination [1–3]. Preexisting pathologies of the lymphatic system such as lymphedema may therefore be identified as a possible contraindication for the use of mRNA vaccines.

Lymphedema is a common condition caused by several different pathomechanisms that remain incompletely understood and can be classified into primary and secondary lymphedema. Secondary lymphedema is an acquired condition, most frequently caused by oncologic surgery such as lymphadenectomy. Primary lymphedema on the other hand, is a rare form of lymphedema which is not directly attributable to another medical condition or treatment. Besides chronic pain, patients suffering from lymphedema usually need to adhere to strict compression therapy and perform lymphatic drainage frequently to manage their symptoms [4–7].

Over the last decades, lymphatic reconstructive surgery has become the gold standard for treating lymphedema if inadequately controlled by conservative treatment. Free lymphatic tissue transfer (VLNT) and lympho-venous anastomoses (LVA) are the cornerstones of lymphatic reconstructive surgery, though they may be combined with the liposuction of fibrotic adipose tissue for immediate volume reduction of the affected anatomical region [8,9].

We herein present the case of a patient who had undergone surgery for primary lymphedema of the lower extremities by using lymph node transfer from the right thoracic wall. Six months later, the patient received a Covid-19-vaccine (two vaccinations) and then developed secondary lymphedema of the upper right arm. In this context, we discuss the potentially increased vulnerability of an impaired lymphatic system due to primary lymphedema and after harvest of lymphatic tissue in the proximity.

## Material and methods

This case report was written in line with the STROBE guidelines. Written consent has been obtained from the patient for the use of imaging material. A 40-year-old female presented with primary lymphedema of the lower extremities starting at the age of 15. Prior patient history was unremarkable, particularly regarding family history of primary lymphedema. Symptoms were more severe in the left leg and conservative therapy did not adequately alleviate her symptoms. Therefore, VLNT from the right thoracic wall to the left groin as well as five LVAs on the left leg were performed. Preoperative lymph-scintigraphy was conducted to mark the lymph nodes draining her right arm with technetium (Tc99) for intraoperative reverse mapping. Reverse lymph node mapping was performed by additional indocyanine green (ICG) injection at the lateral thoracic wall. ICG-positive lymphatic tissue containing lymph nodes from the lateral thoracic wall was harvested, while Tc99 positive lymph nodes were spared to preserve lymphatic drainage of the arm.

The patient received Clindamycin 300 mg 2x/d for seven days to treat a thrombophlebitis of the left leg shortly after surgery. The remaining postoperative follow up was uneventful. At six-weeks follow-up the patient noted a significant tissue softening. Circumference measurements and calculation by the Kuhnke method confirmed a volume reduction of 600 ml.

**Figure 1.**
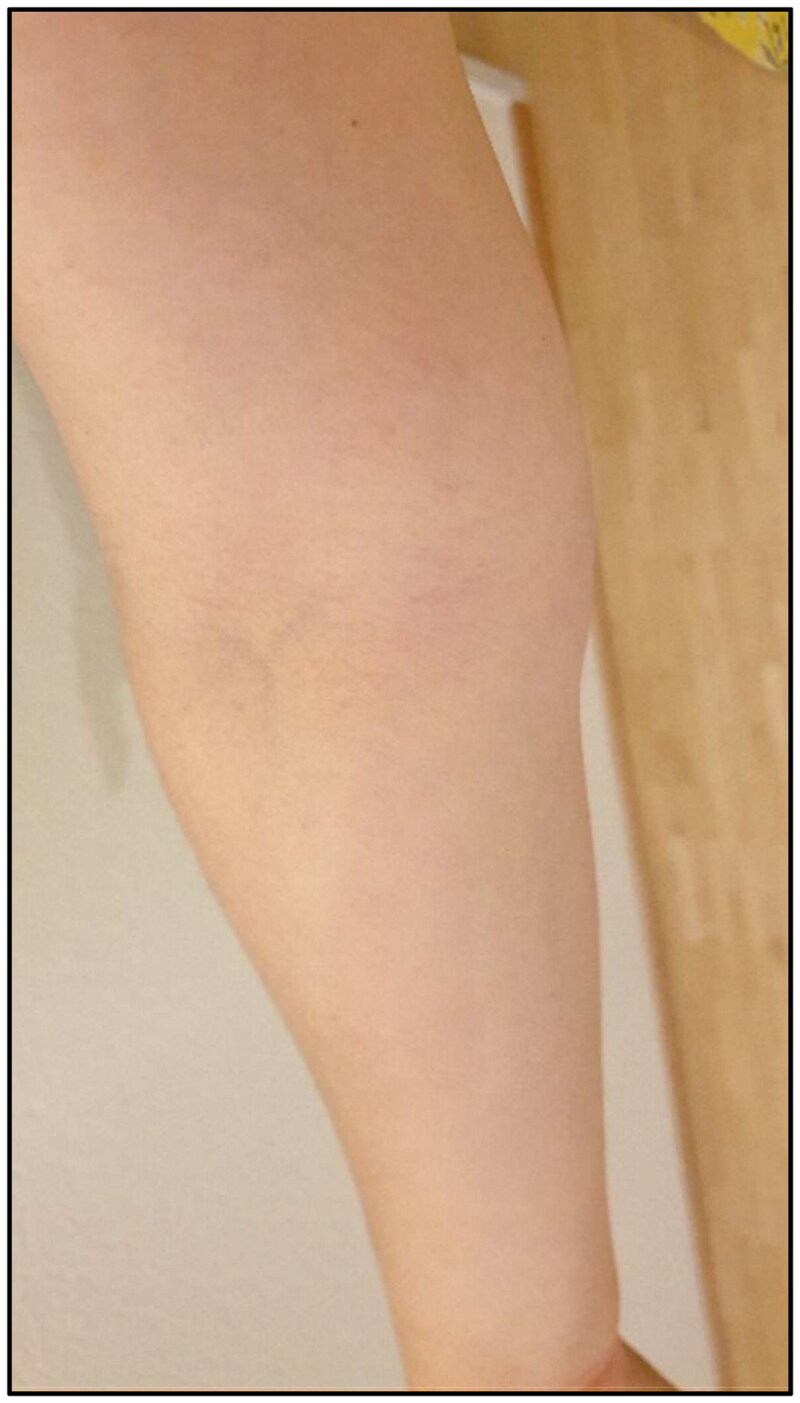
Clinical presentation of red streak on the volar side of the forearm several days after second dose of Covid-19 vaccination.

**Figure 2.**
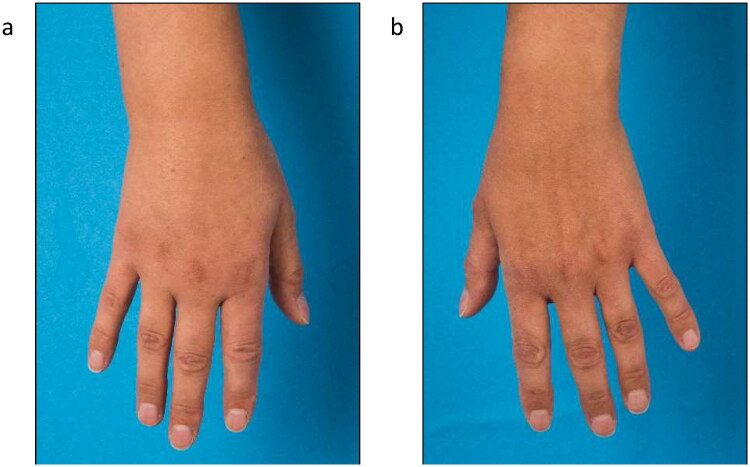
Lymphedema of the right forearm and back of the hand at initial presentation (a) right hand, (b) left hand).

**Figure 3.**
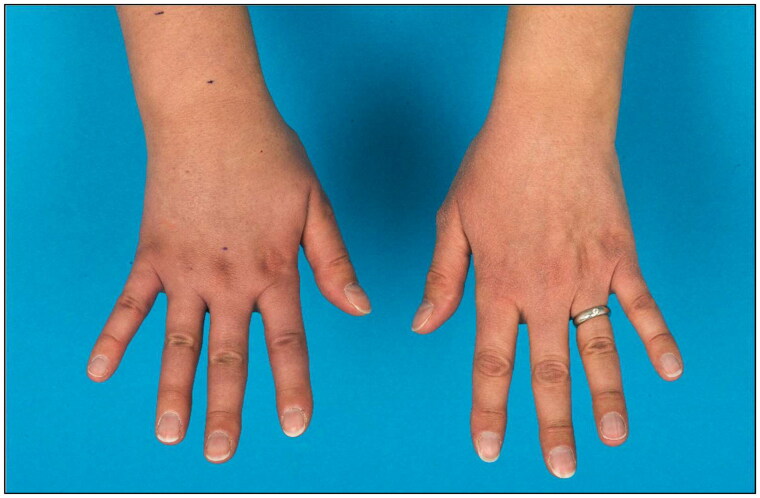
Follow- up after 8 months showed complete regression of the edema at the wrist and only a minor lymphedema of the back of the hand.

Five months postoperatively the patient was vaccinated with mRNA COVID-19 vaccine (Spikevax®) into the left arm with a second dose of Spikevax® being also administered into her left arm one month later. Initially she experienced mild side effects like muscle pain and shaking. After a brief symptom-free interval tenseness of the right arm and a mild swelling at the wrist with the occurrence of a red streak on the volar side of the forearm occurred (Figure 1). Three weeks after the second vaccination the patient was diagnosed with secondary lymphedema of the right arm, with a volume difference of 150 ml compared to the contralateral side (Figure 2). The swelling was clinically evident, accompanied by an increased palpable tenderness of the skin and slight pitting on palpation. Manual lymph drainage and adjustment of personalized compression garments for the right arm were initiated. At eight-months follow-up, regression of the edema could be observed under consistent conservative treatment (Figure 3).

**Figure 4.**
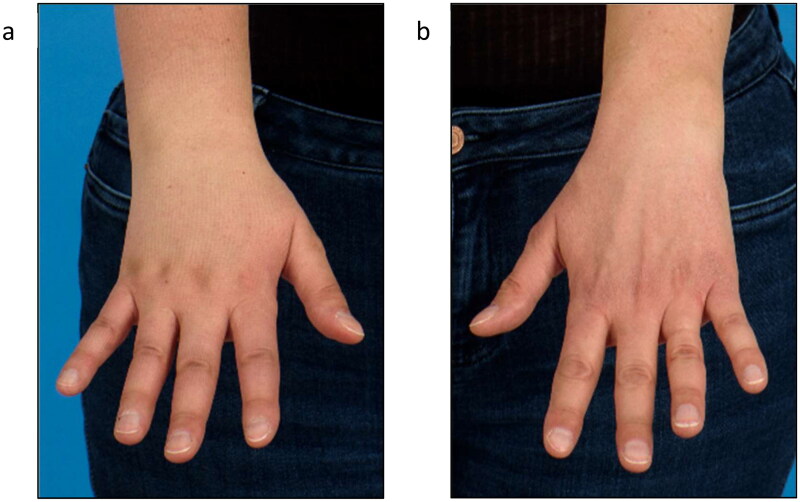
Follow-up 2 years after surgery and 1.5 years after initial diagnosis of lymphedema of the right arm (a) right hand, (b) left hand).

At 2 years follow-up after VLNT the patient presented with a favorable stable postoperative outcome with respect to the left leg. The patient was able to reduce compression stocking class from III to II as well as the frequency of lymph drainage sessions. Swelling and tenderness of the skin decreased as well. Regarding the lymphedema of the right arm, the patient showed initially a volume excess of 37% compared to the left arm. With conservative treatment, relative volume excess could be reduced to 28%. The dorsal hand tended to swell in particular while the lymphedema of the upper arm completely recovered (Figure 4). In addition, lymphoscintigraphy showed a slightly impaired lymphatic drainage with present axillary nodes of the right arm supporting the clinical finding of lymphedema.

## Discussion

We present the case of a patient with a history of primary lymphedema treated by VLNT from the right thoracic wall, who developed secondary lymphedema of the right arm after the second dose of the mRNA 1273 vaccine (Spikevax®). MRNA vaccines are a new type of vaccine and their use increased significantly over a relative short time. On this day these vaccinations are considered safe [2]. As of February 2023, there have been reports of non-severe adverse events in 0.1% of the cases and severe adverse events in 0.04% in the context of vaccination with mRNA 1273 in Switzerland according to the Swiss Agency of Therapeutic Products [1]. Besides the most common side effects such as pain at the injection site, fatigue, headache, myalgia, arthralgia, chills and nausea, swelling and pain of the axillary lymph nodes, ipsilateral lymphadenopathies have been described [1,2]. With respect to the lymphatic system Masayuki states in his paper that mRNA vaccines are encapsulated within lipid nanoparticles of approximately 100 nm in diameter which is why they are transported primarily within the lymphatic system. This possibly leads to local lymph node reactions which is also documented by imaging studies with PET-CT and ultrasound [10,11].

To the knowledge of the authors, nine cases have been reported so far discussing the interaction between COVID-19 vaccines in general and lymphedema. Some authors report lymphedema as an adverse event of the vaccine, others describe specific complications in lymphedema patients after vaccination, such as cellulitis (also see Table 1) [12–15].

**Table 1.** Overview to the reported cases discussing the interaction between COVID-19 vaccines in general and lymphedema.

	Age	Sex	Prior Primary/ secondary	Surgery (LVA)	Type of vaccination	Complication	Onset of complication	Study
1	45	female	Secondary, after cancer surgery	None	mRNA-1273 Spikevax	Cellulitis	After 2nd dose	Okazaki et al.
2	52	female	Secondary, for 7 years as an adverse effect of docetaxel	None	mRNA-1273 Spikevax	cellulitis four times in five months	21 days after 1st dose, 2nd, 3th and 4th month after 2nd dose	
3	49	female	Secondary, lower left limb, lymphedema for 8 years after cancer surgery	LVA (multiple times)	mRNA BNT162b2	Cellulitis	Same day of the 2nd dose	
4	52	female	Secondary after cancer surgery, lower left limb	LVA (multiple times)	mRNA BNT162b2	Cellulitis	11 days after 1st dose	
5	79	female	None	None	mRNA BNT16b2	Lymphedema lower legs	After 2nd dose, time interval unknown	Chung et al.
6	79	male	None	None	mRNA BNT162b2	Lymphedema		
7	68	female	None	None	Astra Zeneca	Lymphedema		
8	68	male	None	None	Sinopharm	Lymphedema lower limbs bilateral and cellulitis	5 days after second dose	Hosseinzadeh et al.
9	45	female	Unknown	None	Vaxzevria	lymphedema left upper limb	10 days after 1st dose	Aimo et al.

In three of these cases other types of vaccinations were applied, in four cases BNT162b2 and in two cases mRNA 1273. Four of these cases were patients with secondary lymphedema who developed cellulitis after vaccination. In four cases lymphedema was described as a transient complication after vaccination in individuals without lymphedema in their prior history. Therefore, there is no reported clinical case with the background of primary lymphedema, nor immediate vascularized lymph node tissue transfer in patient’s history.

Due to the use of the reverse mapping technique, VLNT is considered a relatively safe procedure, which improves clinical findings and quality of life [8,16,17]. According to our own data no incidence of donor-site lymphedema was observed in 37 consecutive patients who received lymphatic tissue harvest from the thoracic wall or the groin, except for the presented case. However, in order to completely avoid the risk of donor site lymphedema the internal protocol has changed to harvesting lymphatic tissue from the omentum over the past years. Even though some studies evaluating the safety of VLNT did not report secondary lymphedema at the donor site [18], Viitanen et al. reported in six out of 10 patients minor changes in lymphatic flow of the donor-site limbs evaluated by lymphoscintigraphy [19]. Considering this, we cannot rule out an impairment of the lymphatic flow at the donor site as predisposing factor. Taking into account that the patient had not only suffered from primary lymphedema for over two decades, but also received VLNT, one may speculate that her lymphatic system may have been impaired at the moment of vaccination.

Many aspects in literature indicate a close relationship between mRNA vaccines and the lymphatic system. It may therefore be assumed, that an additional stressor on the lymphatic system, such as the mRNA vaccination, could provoke this kind of complication. However, it cannot completely be ruled out that any vaccine as a provocative inflammatory agent could have caused a similar reaction.

In conclusion, we report on a case with lymphedema of the right arm after Covid-19 Spikevax® vaccination with a history of primary lymphedema and surgical treatment with VLNT from the axilla to the left groin. Taking current literature into consideration, the indication and timing for mRNA vaccinations in lymphedema patients may be more delicate than in healthy individuals. As a prospective hypothesis it has to be discussed whether lymphedema patients are a population at risk with respect to mRNA vaccines. Further research should determine whether this type of vaccination can safely be used in lymphedema patients or if pathologies of the lymphatic system may be even regarded as a (relative) contraindication.
